# Regulation of epithelial barrier function by Crohn's disease associated gene PTPN2

**DOI:** 10.1186/1753-6561-6-S4-O41

**Published:** 2012-07-09

**Authors:** R Visser, KE Barrett, DE McCole

**Affiliations:** 1 Radboud University Medical Centre, Nijmegen, The Netherlands; 2 University of California, San Diego, USA

## Introduction

The aim of this study was to determine whether there is a synergistic disrupting effect between IFN and TNF on intestinal epithelial barrier function as well as whether this effect is influenced by the knockdown of Crohn's disease associated PTPN2 gene.

## Methods

T84 cells that were either nontransfected or stably transfected with PTPN2 were cultured with IFN and TNF and changes in transepithelial resistance , fluorescein isothiocyanate dextran flux, immunofluorescence and expression of the tight junctional protein claudin-2 and the regulatory protein PTPN2 were assayed.

## Results

Our data demonstrated a decrease in barrier integrity, as measured by TER, by IFN pre-treatment followed by addition of TNF on T84 cell monolayers. This reduction was significantly more dramatic than in controls (***, p<0.001) or T84 cell monolayers treated with IFN- γ alone (*, p<0.05). Furthermore, IFN pre-treatment followed by addition of TNF on T84 cell monolayers demonstrated an alteration in the distribution of claudin-2. PTPN2 showed enhanced expression, as well as a shift in distribution away from the nucleus after this treatment regime. Knockdown of the PTPN2 gene in T84 cell monolayers enhanced the cytokine induced changes as regards to TER. A trend towards increased paracellular permeability as measured by FITC-dextran flux was also observed in PTPN2 deficient T84 cell monolayers treated with IFN alone compared to controls. A higher expression of claudin-2 was observed while experimenting on T84 cell monolayers with repressed PTPN2 expression.

## Conclusions

There is a synergistic disrupting effect between IFN and TNF on intestinal epithelial barrier function. This effect seems to become more dramatic by the knockdown of Crohn's disease associated PTPN2 gene, but further research is needed to elucidate the role of PTPN2 in IBD pathogenesis.

